# 104. Association of Oseltamivir use with Clinical Outcomes of Children Hospitalized with Influenza — Influenza Hospitalization Surveillance Network, 2014-2023

**DOI:** 10.1093/ofid/ofaf695.039

**Published:** 2026-01-11

**Authors:** Kacie Rytlewski, Angela Dunn, Alissa O’Halloran, Jennifer Whitmill Habeck, Isaac Armistead, Nisha B Alden, Pam Daily Kirley, James Meek, Kyle P P Openo, Patricia A Ryan, Sue Kim, Ruth Lynfield, Yomei Shaw, Bridget J Anderson, Maria Gaitan, Krista Lung, Melissa Sutton, H Keipp Talbot, Emma Mendez-Edwards, Jessica R Cataldi, Samuel R Dominguez, Catherine Bozio, Suchitra Rao

**Affiliations:** University of Colorado School of Medicine and Children's Hospital Colorado, Centennial , CO; University of Colorado School of Medicine and Children’s Hospital Colorado, Aurora, Colorado; CDC, Atlanta, GA; Goldbelt Inc, Alpharetta, Georgia; Colorado Department of Public Health and Environment, Denver, Colorado; Colorado Department of Public Health and Environment, Denver, Colorado; California Emerging Infections Program, Oakland, California; Connecticut Emerging Infections Program, Yale School of Public Health, New Haven, Connecticut; Emory University School of Medicine, Atlanta, Georgia; Maryland Department of Health, Baltimore, Maryland; Michigan Department of Health and Human Services, Lansing, Michigan; Minnesota Department of Health, St. Paul, MN; New Mexico Department of Health, Santa Fe, NewMexico; New York State Department of Health, Albany, New York; Rochester Emerging Infections Program, University of Rochester Medical Center, Rochester, New York; Ohio Department of Health, Columbus, Ohio; Public Health Division, Oregon Health Authority, Portland, Oregon; Vanderbilt University Medical Center; Salt Lake County Health Department, Salt Lake City, Utah; University of Colorado School of Medicine, Aurora, Colorado; University of Colorado School of Medicine, Aurora, Colorado; CDC, Atlanta, GA; University of Colorado School of Medicine, Aurora, Colorado

## Abstract

**Background:**

Antiviral treatment has been shown to lower risk of intensive care unit (ICU) admission in children with influenza, though data are limited. National U.S. guidelines recommend antiviral treatment for all hospitalized patients with influenza; however, use has declined since 2019-20, particularly in pediatrics. Our objective was to assess the association between oseltamivir receipt and clinical outcomes among pediatric influenza-associated hospitalizations.

**Figure 1.**
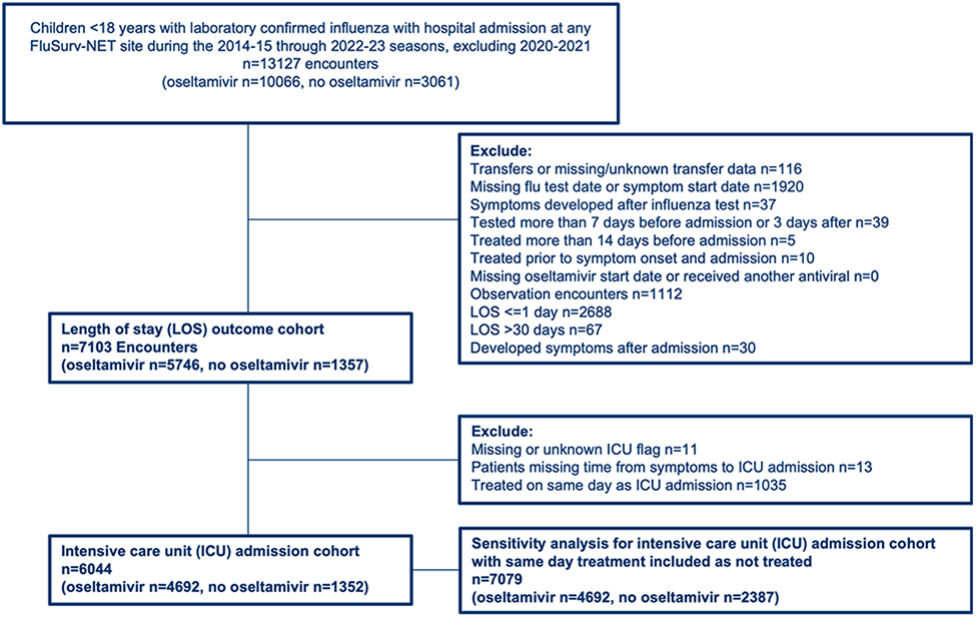
Flow Chart of children with laboratory-confirmed influenza hospitalizations within FluSurv-NET

**Table 1:**
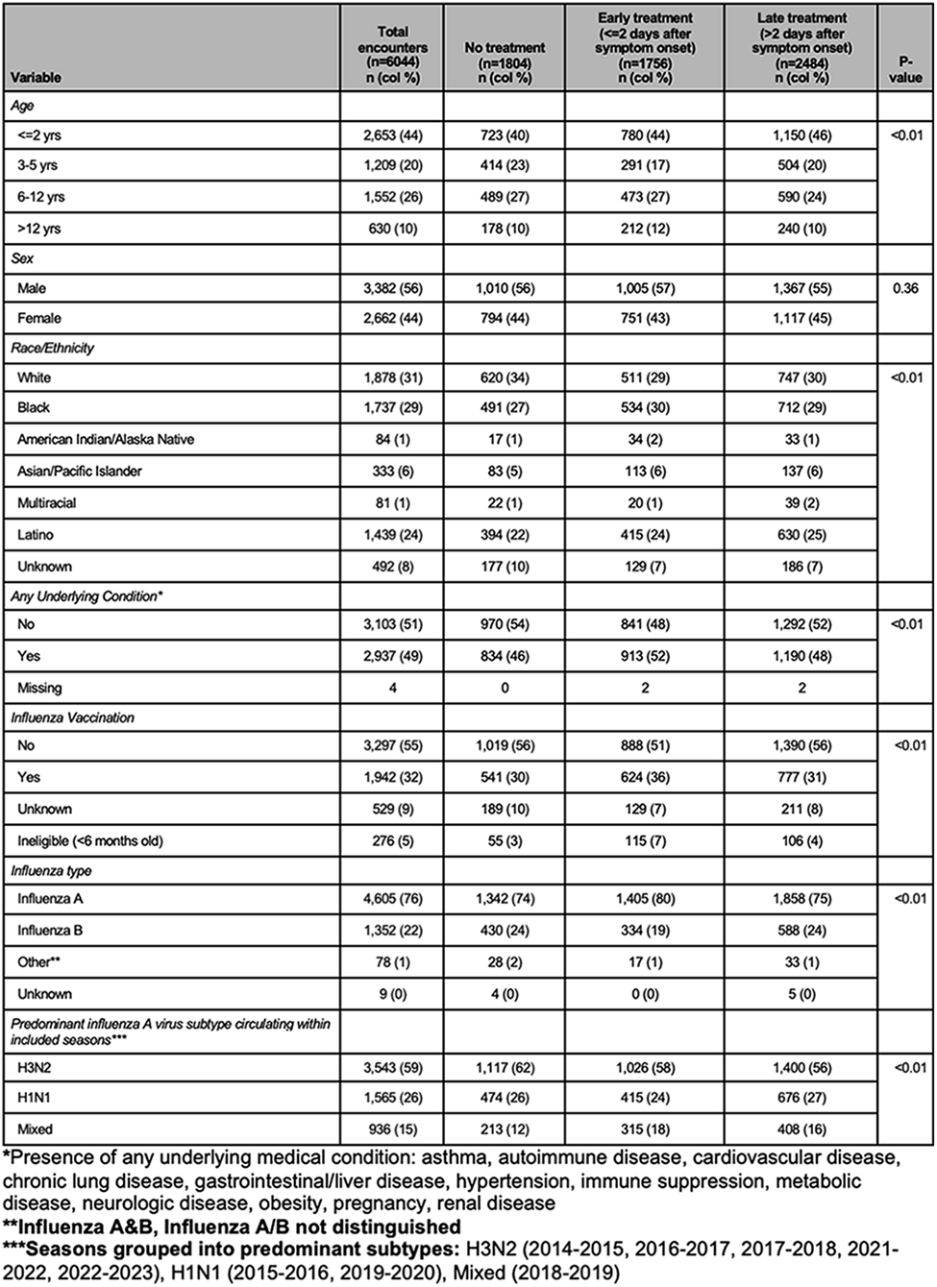
Patient Characteristics, Overall and Stratified by Oseltamivir Receipt and Timing for ICU analysis

**Methods:**

This retrospective cohort study used data from FluSurv-NET, which conducts population-based seasonal surveillance for laboratory-confirmed influenza hospitalizations for all ages across 13 US states. Surveillance staff completed chart abstractions using a standard case report form. We included children < 18 years old hospitalized with influenza during 2014/2015-2022/2023. Adjusted Cox proportional hazard models with oseltamivir receipt as a time-dependent exposure were used to estimate the hazard of intensive care unit (ICU) admission and hospital length of stay (LOS) (see table 2 and 3 for time variable definitions). Covariates for model adjustment were selected from univariate Cox regression analysis (p< 0.2) or a priori.

**Table 2:**
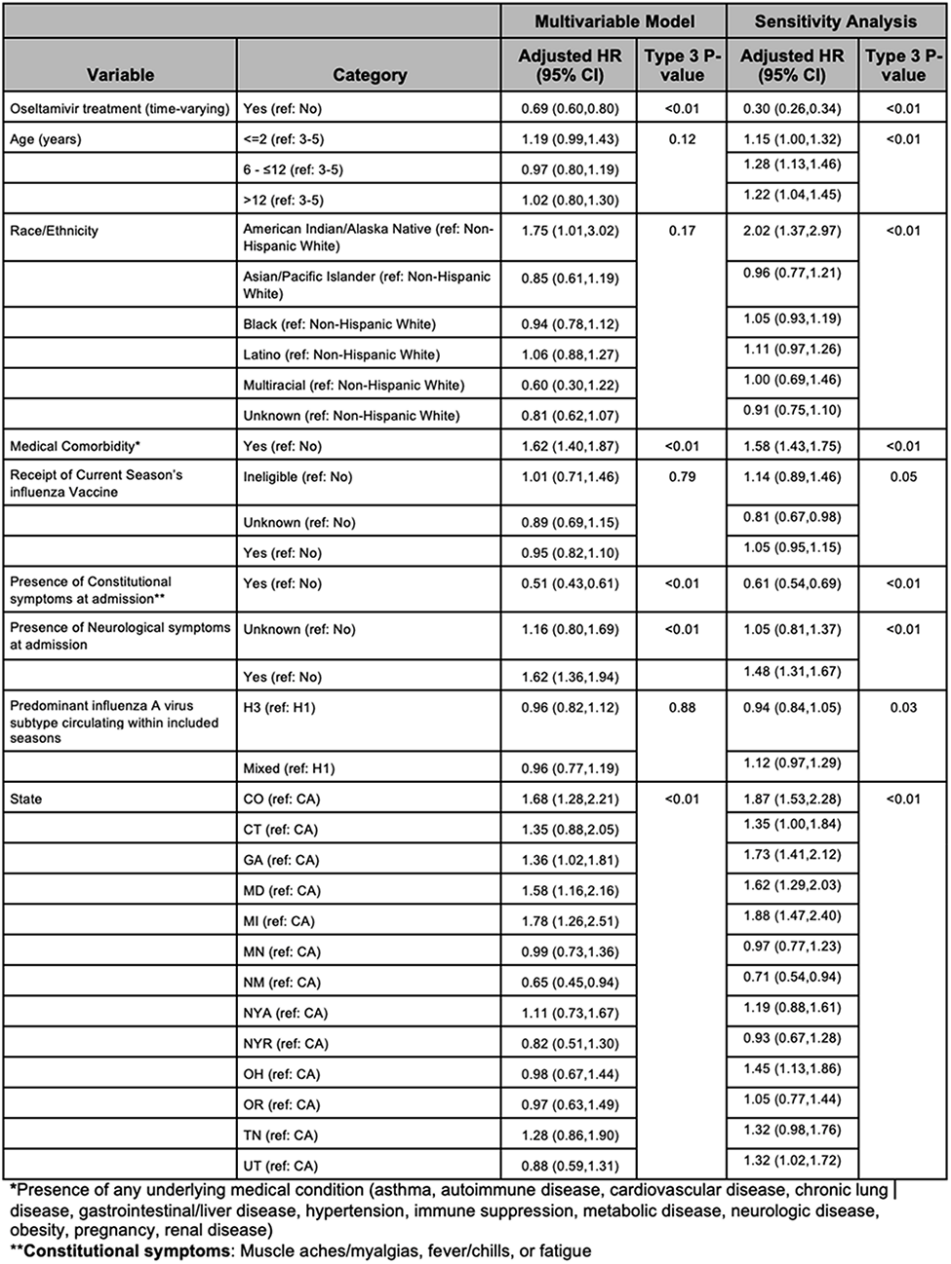
Adjusted Cox Models of Oseltamivir Receipt on Hazard of Intensive Care Unit (ICU) Admission

Multivariate model with adjusted hazard ratios and p-values for ICU outcome (time from symptom onset to ICU admission with patients censored at discharge). Covariates selected from univariate Cox regression analysis with hazard ratio p-value <0.2 include medical complexity, neurological symptoms, constitutional symptoms, and state. A priori covariates include race/ethnicity, predominant influenza A virus subtype circulating within included seasons, influenza vaccination status, and age. Sensitivity analysis performed classifying patients with same-day treatment as ICU admission as not treated.

**Table 3:**
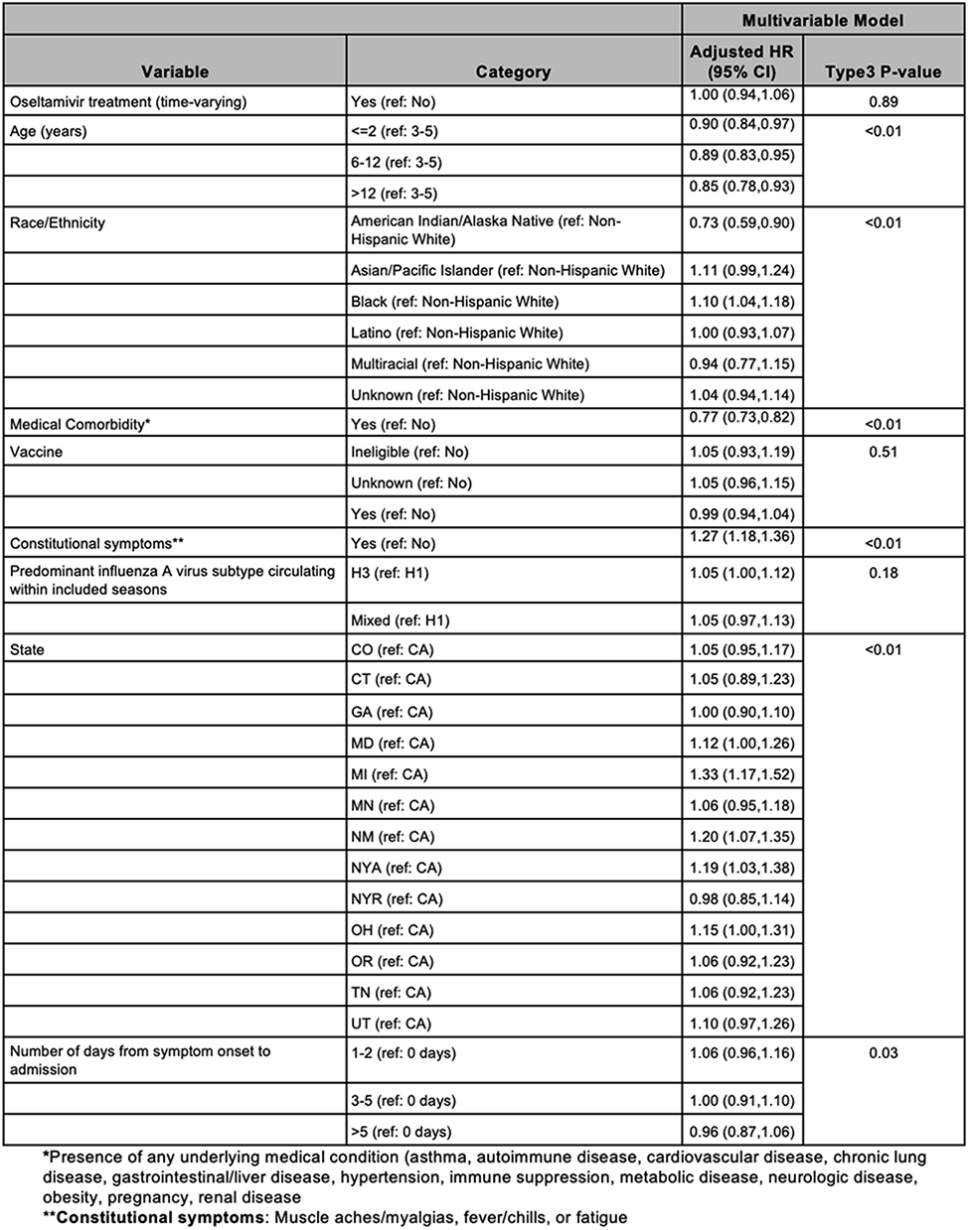
Adjusted Cox Model for Oseltamivir Receipt on Hazard of Hospital Discharge

Multivariate model with adjusted hazard ratios and p-values for LOS outcome (time from admission to discharge). Covariates selected from univariate Cox regression analysis with hazard ratio p-value <0.2 include medical complexity, constitutional symptoms, and state. A priori covariates include race/ethnicity, seasons grouped by predominant influenza A subtype circulating within included seasons, influenza vaccination status, age, and number of days from symptom onset to admission. Thirty-two patients were censored at time of death.

**Results:**

After applying exclusions (Figure 1), 7103 children were included in the LOS analysis (80.9% of whom received oseltamivir) and 6044 in the ICU analysis (of whom 77.6% received oseltamivir). In the ICU analysis, the median age was 3 years, 56% were male, 31% were non-Hispanic (NH) white, 29% were NH black and 49% had medical comorbidities (Table 1). In adjusted models, oseltamivir treatment reduced the hazard of ICU admission (aHR 0.69, 95% CI 0.60-0.80), compared with untreated children. A sensitivity analysis including those treated on the same day as ICU admission categorized as not treated strengthened this association (aHR 0.30, 95% CI 0.26-0.34). Treatment did not have a significant impact on the hazard of time to discharge (aHR 1.00, 95% CI 0.94-1.06) (Tables 2 and 3).

**Conclusion:**

In this national cohort of children hospitalized with influenza, oseltamivir treatment significantly reduced the hazard of ICU admission by 31%, but did not impact hospital LOS. These findings support current national guidelines to treat children hospitalized with influenza with oseltamivir.

**Disclosures:**

All Authors: No reported disclosures

